# Intraoperative laparoscopic detection of sentinel lymph nodes with indocyanine green and superparamagnetic iron oxide in a swine gallbladder cancer model

**DOI:** 10.1371/journal.pone.0248531

**Published:** 2021-03-11

**Authors:** Kisyo Mihara, Sachiko Matsuda, Yuki Nakamura, Koichi Aiura, Akihiro Kuwahata, Shinichi Chikaki, Masaki Sekino, Moriaki Kusakabe, Shunichi Suzuki, Daiichiro Fuchimoto, Akira Onishi, Junko Kuramoto, Kaori Kameyama, Osamu Itano, Hiroshi Yagi, Yuta Abe, Minoru Kitago, Masahiro Shinoda, Yuko Kitagawa

**Affiliations:** 1 Department of Surgery, Kawasaki Municipal Kawasaki Hospital, Kawasaki-ku, Kawasaki, Kanagawa, Japan; 2 Department of Surgery, Keio University School of Medicine, Shinjuku-ku, Tokyo, Japan; 3 Graduate School of Engineering, The University of Tokyo, Bunkyo-ku, Tokyo, Japan; 4 Graduate School of Agricultural and Life Sciences, Research Center for Food Safety, The University of Tokyo, Bunkyo-ku, Tokyo, Japan; 5 Matrix Cell Research Institute, Inc., Ushiku, Ibaraki, Japan; 6 Division of Animal Sciences, Institute of Agrobiological Sciences, National Agriculture and Food Research Organization (NARO), Tsukuba, Ibaraki, Japan; 7 Laboratory of Animal Reproduction, Department of Animal Science and Resources, College of Bioresource Sciences, Nihon University, Fujisawa, Kanagawa, Japan; 8 Department of Pathology, Keio University School of Medicine, Shinjuku-ku, Tokyo, Japan; 9 Department of Diagnostic Pathology, Keio University Hospital, Shinjuku-ku, Tokyo, Japan; 10 Department of Gastrointestinal Surgery, International University of Health and Welfare, Chiba, Japan; Brandeis University, UNITED STATES

## Abstract

Mapping of sentinel lymph nodes (SLNs) can enable less invasive surgery. However, mapping is challenging for cancers of difficult-to-access visceral organs, such as the gallbladder, because the standard method using radioisotopes (RIs) requires preoperative tracer injection. Indocyanine green (ICG) and superparamagnetic iron oxide (SPIO) have also been used as alternative tracers. In this study, we modified a previously reported magnetic probe for laparoscopic use and evaluated the feasibility of detecting SLNs of the gallbladder using a laparoscopic dual tracer method by injecting ICG and SPIO into five swine and one cancer-bearing swine. The laparoscopic probe identified SPIO nanoparticles in the nodes of 4/5 swine *in situ*, the magnetic field counts were 2.5–15.9 μT, and fluorescence was detected in SLNs in all five swine. ICG showed a visual lymph flow map, and SPIO more accurately identified each SLN with a measurable magnetic field quite similar to the RI. We then developed an advanced gallbladder cancer model with lymph node metastasis using recombination activating gene 2-knockout swine. We identified an SLN in the laparoscopic investigation, and the magnetic field count was 3.5 μT. The SLN was histologically determined to be one of the two metastatic lymph nodes. In conclusion, detecting the SLNs of gallbladder cancer *in situ* using a dual tracer laparoscopic technique with ICG and SPIO was feasible in a swine model.

## Introduction

Application of the sentinel lymph node (SLN) theory is beneficial because it can result in less invasive surgery for many cancers, such as skin [1], breast [2], gastrointestinal [3] and gynaecological cancers [4], even during laparoscopic operations [5]. The standard approach for detecting SLNs is the dual tracer method using radiolabelled tin colloid and blue dye [6]. The radioisotope (RI) tracer is injected 2 to 24 hours prior to surgery for accurate SLN detection [7], which limits the application of the SLN theory to organs that are easily accessible from the surface of the body or by endoscopy.

The clinical significance of SLNs in patients with gallbladder cancer has not yet been clearly determined. The reported incidence of lymph node metastasis is 10.9% or less in patients with early gallbladder cancer [8], and the optimal surgical strategy for early gallbladder cancer remains debatable [9]. These patients may benefit from SLN mapping to enable planning of the appropriate extent of lymphadenectomy. However, the gallbladder is one of the most difficult organs to access from the surface of the body or by endoscopy because its wall is particularly thin, and needle punctures can easily cause bile leakage. Furthermore, limitations on preoperative access have prevented the use of traditional RI tracer methods.

Recently, an indocyanine green (ICG) and superparamagnetic iron oxide (SPIO) dual tracer was applied in breast cancer [10]. ICG fluorescence imaging is reportedly a promising tool for SLN detection in patients with breast, gastric [11] and colorectal cancer [12]. With a fluorescence imaging system, lymph flow and SLNs are detected soon after injection, even in dense adipose tissue. However, because the ICG tracer is small, it passes through to downstream lymph nodes, making it difficult to quantitatively analyse SLNs, which is a disadvantage of this technique [13]. SLN detection using SPIO nanoparticles has been reported by Douek *et al*. [14]. The magnetic tracer was taken up by macrophages in the lymph nodes and detected by a handheld magnetometer [14]. In a previous study, it was shown that SPIO reaches the axillary lymph nodes within minutes after injection into the breast [15].

To detect SPIO, several magnetometers have been developed [14,16–19]. We also previously developed a handheld magnetic probe that consists of a Hall sensor and a permanent magnet [20]. Clinical tests using SPIO and blue dye tracers in patients with breast cancer have shown that the handheld magnetic probe was useful for detecting SLNs containing the magnetic nanoparticles [21].

Swine provide an appropriate model for measuring lymphatic flow and are useful for visceral preclinical surgical simulations because their anatomy and size are similar to that of humans, and they have been widely used in endoscopic and surgical training [22]. In addition, we previously established a lymph node metastasis model of a subcutaneous tumour using recombination activating gene 2 *(RAG2)*-knockout (KO) immunodeficient swine [23] to evaluate the cancer-bearing circumstance that closely reflects the clinical situation.

The aim of this study was to evaluate the feasibility of detecting SLNs of gallbladder cancer using an ICG and SPIO dual tracer *in situ* during laparoscopic surgery. We modified the previously reported magnetic probe [20] for use in laparoscopic surgery in this study.

## Material and methods

### SPIO nanoparticles

The SPIO used was a ferucarbotran (44.6 mg of iron in a 1.6-mL vial) purchased from Fujifilm Toyama Chemical (Tokyo, Japan). Ferucarbotran has a carboxydextran shell of approximately 60 nm to stabilise magnetic nanoparticle aggregations [24].

### Preparation of the dual tracer for SLN detection

ICG (25 mg; Diagnogreen; Daiichi Sankyo, Tokyo, Japan) was diluted in 10 mL of water. The mixed tracer for SLN detection consisted of 0.8 mL ferucarbotran, 0.2 mL ICG and 1 mL saline (2 mL total volume).

### Cell lines

The human skin epithelial carcinoma cell line A431 characterized by high epidermal growth factor receptor expression was obtained from the American Tissue Culture Collection, (ATCC® CRL1555™, Rockville, MD, USA). A431 cells were transfected with green fluorescent protein (GFP) and used for the gallbladder cancer model. The cells were maintained in Dulbecco’s modified Eagle’s medium with 10% heat-inactivated foetal bovine serum (Thermo Fisher Scientific, Waltham, MA, USA) in a humidified 5% (v/v) CO_2_ incubator at 37°C. Mycoplasma testing was performed before experiments.

### Animals

Six crossbred swine (Landrace × Yorkshire × Duroc; also referred to as wild-type) were purchased from the National Federation of Agricultural Cooperative Associations (Tokyo, Japan). One swine underwent open surgery, and five swine underwent laparoscopic surgery to detect SLNs when they weighed 24.5 ± 1.9 kg (Table 1). The surgery was performed one week after acclimation. All six swine were sacrificed by an intravenous potassium chloride injection during deep respiratory anaesthesia at the end of surgery. No animals met the humane endpoint criteria before surgery.

**Table 1:** All experimental data for swine.

	1	2	3	4	5	6	7
Genotype	wild	wild	wild	wild	wild	wild	RAG2-KO
Body weight (kg)	22.8	27.0	23.0	26.4	24.6	23.0	13.0
Open surgery	+	-	-	-	-	-	-
Laparoscopic surgery	-	+	+	+	+	+	+
Tracer injection (ml): ICG + ferucarbotran	0+0.8	0.2+0.8	0.2+0.8	0.2+0.8	0.2+0.8	0.2+0.8	0.2+0.8
Regional lymph node dissection	+	+	+	+	+	+	+
A431 tumour cell injection	-	-	-	-	-	-	+
Histological examination	+	+	+	+	+	+	+

KO = Knockout, ICG = indocyanine green.

One RAG2-KO [25] swine was produced at the National Agriculture and Food Research Organization for the tumour model. Three weeks was the experimental endpoint due to cancer growth in this model. We considered the ethical endpoints to be anorexia, weight loss and crouching of swine. We assessed the morbidity of swine daily, and their weights were measured weekly. After the surgical procedure, the swine was sacrificed by an intravenous potassium chloride injection during deep respiratory anaesthesia. No animals met the humane endpoint criteria before the end of the experiments or died before meeting the criteria for euthanasia.

All swine were maintained on a standard laboratory chow diet, had free access to tap water and were cared for by specialists of the Laboratory Animals Centre at Keio University. All research staff involved in the animal experiments took the Animal Research Course and Orientation provided by the Laboratory Animals Centre at Keio University.

The Institutional Animal Care and Use Committees of Keio University (Approval number: 8073) approved this study. All animal experiments were performed in accordance with the local ethics laws, the regulations of the local ethics committee and the Institutional Guidelines on Animal Experimentation at Keio University.

### SLN classification

**Fig 1:**
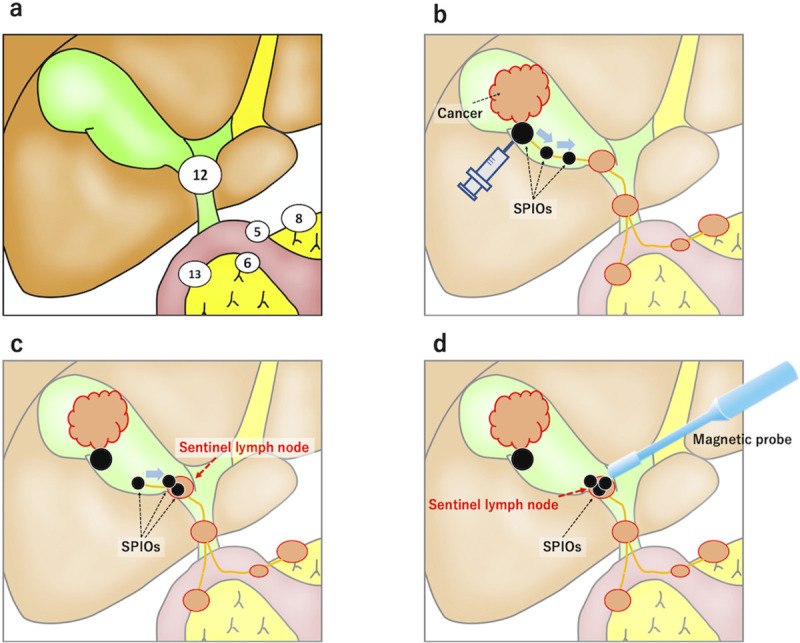
Regional lymph node map for gallbladder cancer and the procedure for detecting sentinel lymph nodes (SLNs) using the magnetic method. (**a**) Japanese classification of the regional lymph nodes in gallbladder cancer. Station number 5, suprapyloric nodes; 6, infrapyloric nodes; 8, nodes along the common hepatic artery; 12, nodes in the hepatoduodenal ligament; and 13, nodes posterior to the pancreatic head. (**b, c, d**) Principles of the procedure based on the magnetic detection of SLNs for gallbladder cancer using the laparoscopic magnetic probe.

To evaluate SLN distribution, the regional lymph nodes were classified according to the simplified Japanese classification for cancer of the biliary tract [26] (Fig 1A). The relationships between the lymph node station number and the anatomical topographical name are as follows: station 5, suprapyloric; 6, infrapyloric; 8, along the common hepatic artery; 12, in the hepatoduodenal ligament; and 13, posterior to the pancreatic head.

### Surgical procedure

All surgeries were performed after premedication with intramuscular medetomidine (0.02 mL/kg) and midazolam (0.1 mL/kg). Anaesthesia was maintained with 2%–2.5% isoflurane after intubation.

In open surgery, SPIO nanoparticles were injected into the gallbladder wall after making a median incision. The SPIO tracer consisting of 0.8 mL ferucarbotran and 1.2 mL saline (total volume 2 mL) was injected using a 27-G needle. The gallbladder and all regional lymph nodes around the gallbladder were extracted after 15 minutes in accordance with a previously reported dye method [27]. Iron deposition was examined by iron quantification and histological examination.

In laparoscopic surgery, four 12-mm trocars were placed in the upper abdomen, and a pneumoperitoneum was established. Two millilitres of the mixed dual tracer were injected into the gallbladder wall using a 27-G needle (Fig 1B), and the dual tracer moved into the lymph nodes via the lymphatic vessels (Fig 1C). Then, the laparoscopic magnetic probe was used to identify the lymph nodes containing SPIO nanoparticles (Fig 1D). Fluorescent signals were observed immediately after injection using a laparoscopic near-infrared (NIR) camera system (Visera Elite II; Olympus, Tokyo, Japan). Fifteen minutes after the injection, the regional lymph nodes were identified and examined with a laparoscopic magnetic probe inserted through the left 12-mm trocar. The SLN examinations were completed within 30 minutes of the injection. Next, all regional lymph nodes were removed, and the magnetic field was measured *ex vivo* using a magnetic probe. Then, iron deposition and histological examinations were performed.

### Magnetic probe

**Fig 2:**
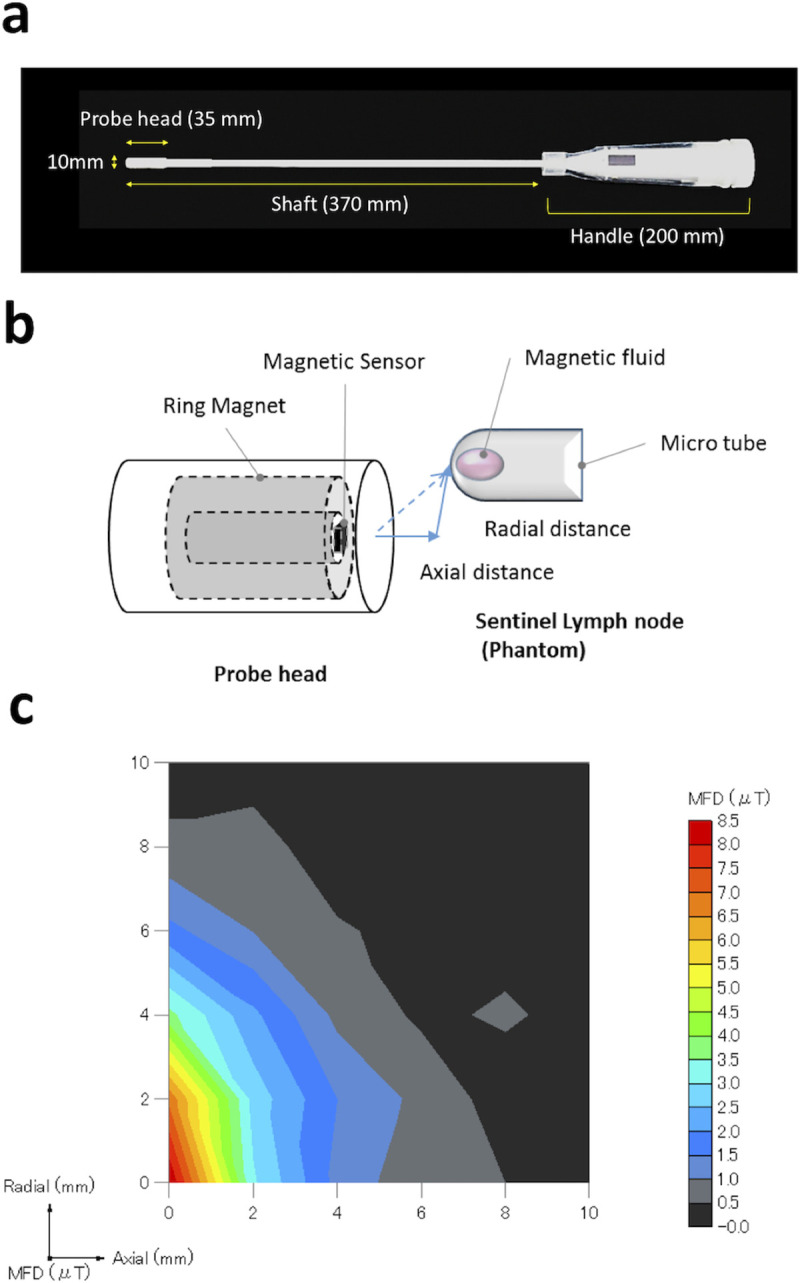
The modified magnetic probe. (**a**) Photograph of the laparoscopic magnetic probe. The display and battery are contained in the handle, which has a sterilisable plastic cover. (**b**) Scheme of the experimental procedure for evaluating the detection range of the laparoscopic magnetic probe. **(c)** Map of the sensor output obtained with a magnetic fluid sample scanned in the radial–axial plane. MFD: magnetic flux density.

A Hall sensor probe developed by our team [20] was modified to generate a cordless laparoscopic probe for this study. The probe consists of a permanent magnet and a short-rod sensor (NHE520, high-output-type Hall element using evaporated indium antimonide film) with the Hall element in the sensor located at the magnetic null point, enabling highly sensitive measurements that are not influenced by the ambient strong magnetic fields from the neodymium magnet. The shaft of the laparoscopic probe is 380 mm long, and the diameter of the laparoscopic head is 10 mm, enabling passage of the probe through standard 12-mm trocars (Fig 2A). The probe runs on batteries located inside its handle; thus, it has no cable and weighs 125 g including the shaft.

### Evaluation of the detection range using a laparoscopic probe

The magnetic material used for this measurement was ferucarbotran, which consists of 280 μg of iron inserted into a microtube. The probe head is fixed, and the magnetic fluid can be displaced using a linear XY stage. The experimental design for measuring the detection range is described in Fig 2B.

### Iron quantification

The amount of iron was measured using a previously described device [28]. Measured values (voltage based on Faraday’s law) were converted into the amount of iron using the coefficient of 118 μg/mV, which was calibrated using a superconducting-quantum-interference device (MPMS-5S; Quantum Design, San Diego, CA, USA).

### Histological analysis

All resected regional lymph nodes were examined histologically after staining of the paraffin-embedded samples with haematoxylin and eosin, Perl’s Prussian blue and immunostaining. Haematoxylin and eosin staining and Perl’s Prussian blue staining for iron were performed in a standard manner. Immunostaining is described below.

### Immunohistochemistry

Specimens were deparaffinised in xylol and rehydrated in a descending series of ethanol concentrations. Antigens were retrieved by incubating the samples in Target Retrieval Solution (Dako Japan, Tokyo, Japan) at 121°C for 10 min. Specimens were incubated with a primary antibody (Dako Japan) against GFP (1:100, ab183734, Dako Japan) overnight at 4°C and then Envision (Dako Japan) for 30 min. Immune complexes were visualised using diaminobenzene for 1 min, and counterstaining with haematoxylin was performed for 1 min.

### RAG2-KO swine gallbladder cancer model with lymph node metastasis

Tumour cells were implanted into RAG2-KO swine during open laparotomy. Using a 27-G needle, A431 cells (1 × 10^7^ in 1 mL phosphate-buffered saline) expressing GFP were injected into the gallbladder wall of the RAG2-KO swine when it was 7 weeks old and weighed 9.2 kg. When this swine was 10 weeks old and weighed 13 kg, laparoscopic surgery was performed to assess tumour formation in the gallbladder and detect SLNs using the dual tracer method.

### Fluorescence microscopy

A stereoscopic fluorescence microscope (SMZ-25; objective lens, SHR Plan Apo 0.5×; Nikon, Tokyo, Japan) with a Nikon DS-Ri2 camera (Nikon) was used for the *ex-vivo* detection of GFP. Images were processed using an NIS-Elements D system (Nikon).

## Results

### Evaluation of the magnetic probe for laparoscopic surgery

Because the working range is restricted by port positions in laparoscopic surgery, the probe head is assumed to contact the target organ diagonally. Therefore, we adjusted the shape of the magnet for laparoscopic use accordingly. Fig 2C presents the relationship between the location of the magnetic material ahead of the probe head and the measured magnetic field. The detection range with the minimum measurable value set at 1 μT was 5 mm longitudinally and 7 mm laterally (Fig 2C).

### Evaluation of iron distribution after SPIO injection in open surgery

**Fig 3:**
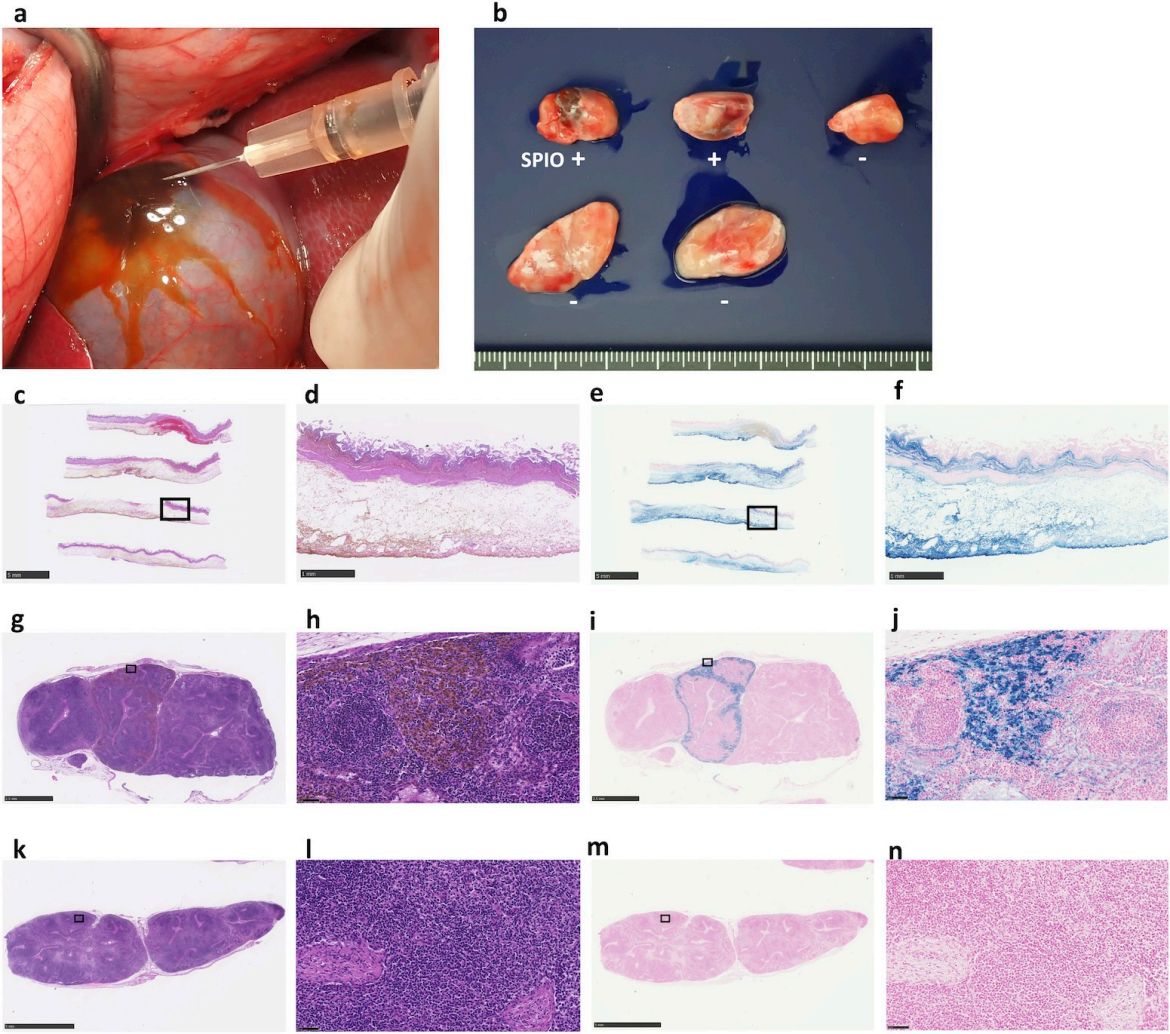
Histological examination of the gallbladder and lymph nodes from wild-type swine in open surgery. (**a**) Injection of the superparamagnetic iron oxide (SPIO) tracer into the gallbladder wall. (**b**) Five resected regional lymph nodes. (**c, d**) Haematoxylin and eosin staining and (**e, f)** iron staining of the gallbladder. (**g, h**) Haematoxylin and eosin staining and (**i, j**) iron staining (Perl’s Prussian blue) of an SPIO-positive sentinel lymph node (SLN). (**k, l**) Haematoxylin and eosin staining and (**m, n**) iron staining of an SPIO-negative lymph node. Scale bar = 5 mm (**c, e, k, m**), 1 mm (**d, f**), 2.5 mm (**g, i**) and 50 μm (**h, j, l, n**).

To explore the possibility of expanding the use of the SLN principle to gallbladder cancer, we evaluated iron distribution from the gallbladder wall (Fig 3A) to the regional lymph nodes (Fig 3B) in wild-type swine by open surgery. There was brownish iron deposition in the gallbladder wall (Fig 3A). By histological examination, iron deposition was observed in the connective tissue of all layers of the gallbladder from the mucosa to the serosa (Fig 3C and 3D), and this distribution was confirmed with iron-stained samples (Fig 3E and 3F). The iron content in the harvested gallbladder was measured using the iron quantification device, and the measurement was 3.83 mg, which represents 17.2% of the initial injection.

We then harvested and examined five regional lymph nodes. Brownish iron deposition was observed in the two positive lymph nodes (Fig 3B), and the iron contents were 438 μg and 138 μg in these lymph nodes. The presence of iron was confirmed by Prussian blue staining microscopically (Fig 3G–3J), and we found that iron was distributed mainly in the marginal sinuses of the lymph nodes (Fig 3J). In contrast, iron deposits were not found in the other three lymph nodes (Fig 3K–3N). The iron content was within a detectable range of the iron quantification device.

### Laparoscopic identification of SLNs in wild-type swine with the ICG and SPIO dual tracer

**Fig 4:**
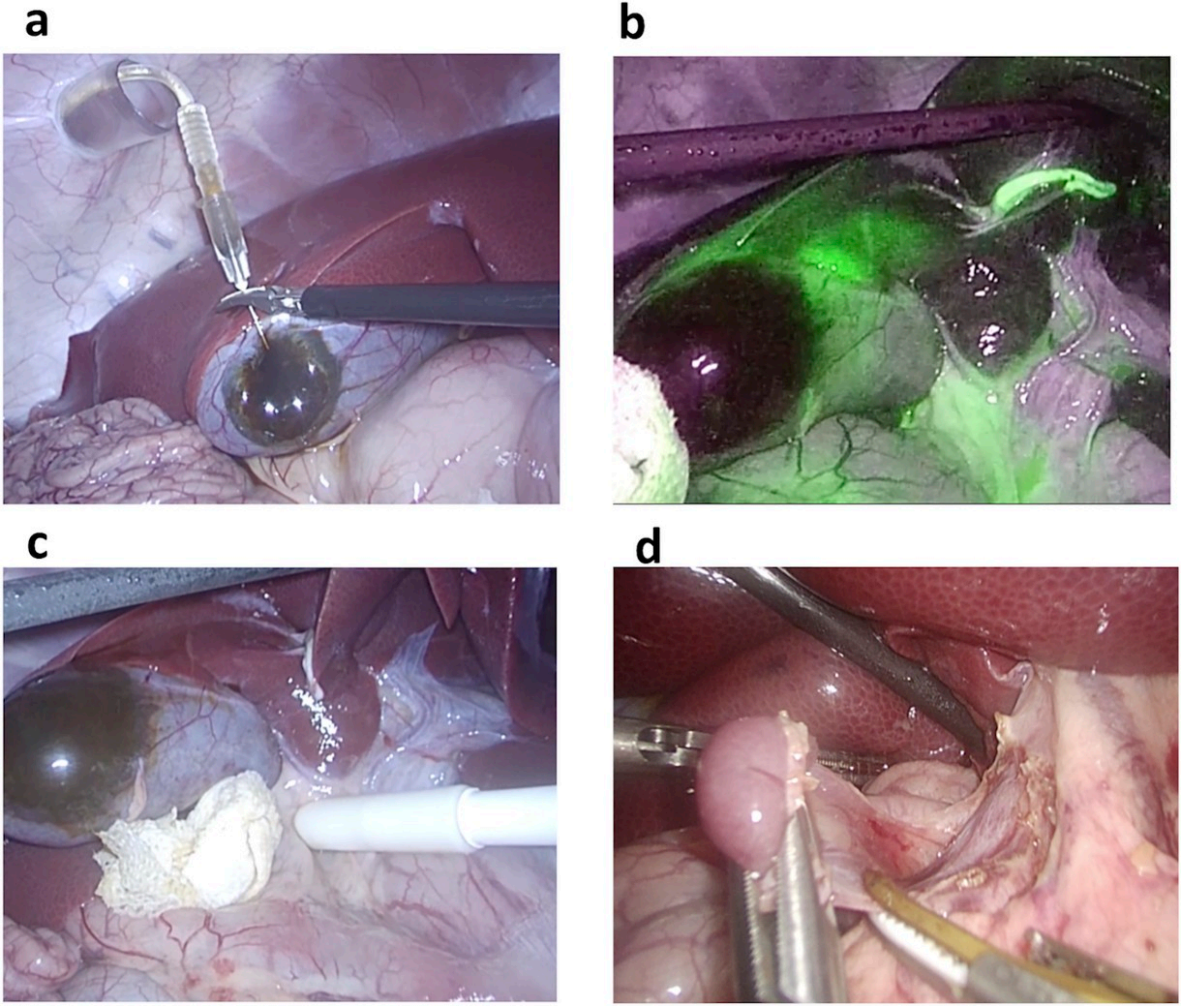
Laparoscopic sentinel lymph node (SLN) detection with a mixed tracer. (**a**) Injection of the mixed tracer into the gallbladder wall. (**b**) Indocyanine green fluorescence signals identified with near-infrared electronic laparoscopy. (**c**) Magnetic field evaluation with the laparoscopic magnetic probe. (**d**) Resection of the regional lymph nodes.

Using laparoscopic surgery, both SPIO and ICG tracers could be injected into the gallbladder intraoperatively under direct vision. We performed laparoscopic surgery on five wild-type swine to detect the SLNs in normal gallbladders using the dual tracer method (S1 Video). In detail, we injected the mixed tracer into the gallbladder wall after inducing a pneumoperitoneum (Fig 4A). Immediately after injection, we detected fluorescence signals intraoperatively in all swine using NIR electronic laparoscopy (Fig 4B). We identified fluorescent hotspots in the lymph nodes, lymphatic vessels and liver parenchyma in the vicinity of the gallbladder. In addition, we observed fluorescent signals in three to four lymph nodes in all five swine (Table 2). We then detected the magnetic field of SPIO signals *in situ* with a laparoscopic magnetic probe 15 minutes after the injection (Fig 4C). The laparoscopic probe reacted only when touching a target lymph node and did not show a shine-through effect. SPIO nanoparticles accumulated in one to two lymph nodes in four of the five swine, and the magnetic field counts were 2.5–15.9 μT in the positive lymph nodes *in situ* (Table 2). We failed to detect a magnetic signal in one of the five swine (Swine 3). The magnetic field counts at the injection site in the gallbladder wall were over the detection rate. After completing the SLN investigations, all regional lymph nodes (Fig 4D) were excised, and the magnetic fields were measured using the same magnetic probe *ex-vivo*. The counts were 8.7–44.1 μT in the positive lymph nodes (Table 2), and the amount of iron was 99.1–440 μg in these nodes (Table 2). All topographical distributions of the SLNs detected by the dual tracer in these five swine are shown in Table 2. The specific SLNs detected by SPIO were lymph node station number 13 in three swine (Swine 1, 4 and 5) and lymph node station number 8 in Swine 2.

**Table 2:** Topographical distribution of sentinel lymph nodes in the five swine.

Node station	Swine case number				
	1	2	3	4	5
#5	N / 0 / 0 / 0	ND	P / 0 / 0 / 0	N / 0 / 0 / 0	N / 0 / 0 / 0
#6	ND	ND	ND	ND	N / 0 / 0 / 0
#8	N / 0 / 0 / 0	P / 4.2 / 15.8 / 147	N / 0 / 0 / 0	N / 0 / 0 / 0	P / 0 / 0 / 0
#12	P / 0 / 0 / 0	P / 0 / 0 / 0	P / 0 / 0 / 0	P / 0 / 0 / 0	P / 0 / 0 / 0
#12	ND	P / 0 / 0 / 0	ND	P / 0 / 0 / 0	ND
#13	P / 3.5 / 44.1 / 307	P / 0 / 0 / 0	P / 0 / 0 / 0	P / 15.9 / 11.1 / 440	P / 9.1 / 10.4 / 184
#13	P / 2.5 / 8.7 / 99.1	ND	ND	ND	N / 0 / 0 / 0

Data are shown in the following sequence: Indocyanine green hotspot (P, positive; N, negative)/magnetic field during laparoscopic surgery (μT)/magnetic field of the extracted lymph nodes (μT)/iron content (μg). ND: Not detected.

### Histological evaluation after laparoscopic surgery

**Fig 5:**
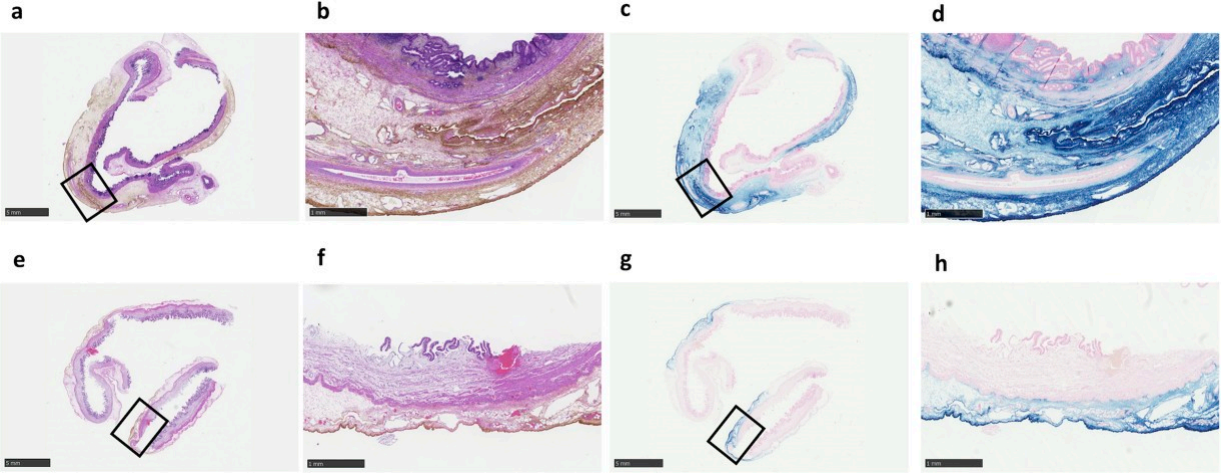
Histological examination of the gallbladder from wild-type swine in laparoscopic surgery. (**a, b**) Haematoxylin and eosin staining and (**c, d**) iron staining (Perl’s Prussian blue) of the gallbladder of swine with a superparamagnetic iron oxide (SPIO)-positive sentinel lymph node (SLN). (**e, f**) Haematoxylin and eosin staining and (**g, h**) iron staining of the gallbladder of the SLN SPIO-negative swine (Swine 3). Scale bar = 5 mm (**a, c, e, g**) and 1 mm (**b, d, f, h**).

We examined all harvested gallbladders and lymph nodes histologically and found that iron was distributed in all SPIO-positive lymph nodes. In the gallbladder, there was brownish iron deposition in the connective tissue of all layers of the gallbladder from the mucosa to the serosa (Fig 5A and 5B), and this distribution was also visible in iron-stained specimens (Fig 5C and 5D). In contrast, iron deposits were limited to the superficial gallbladder layers from the subserosa to the serosa in the SLN-negative swine (Swine 3; Fig 5E–5H).

### Identification of SLNs in the RAG2-KO swine gallbladder cancer model

It has been shown that lymph flow can change in the presence of tumours. Because advanced tumours can theoretically obstruct lymphatic pathways, lymphatic mapping techniques are important in individuals with cancer [13]. To examine the dual tracer and magnetic laparoscopic probe in a situation that more closely reflects that observed clinically, we used a gallbladder cancer swine model with lymph nodes metastases. After laparoscopic peritumoral injection of the mixed dual tracer into the gallbladder in the RAG2-KO swine, we used an NIR electronic laparoscope and a magnetic probe to examine the SLNs *in situ*, and the displayed magnetic field count was 3.5 μT in the positive lymph nodes (Table 3). After laparotomy, we also resected the lymph nodes and examined them *ex vivo* with the same magnetic probe (44.1 μT) and iron quantification apparatus (86.5 μg) to identify iron deposition (Table 3), which we found only in the number 13 lymph node.

**Table 3:** Topographical distribution of the sentinel lymph nodes and metastatic lymph nodes in a RAG2-knockout swine gallbladder cancer model.

Node station	SLN detection	Histological exam
#5	N / 0 / 0 / 0	ND
#8	N / 0 / 0 / 0	lymph node metastasis
#12	P / 0 / 0 / 0	ND
#13	P / 3.5 / 44.1 / 86.5	lymph node metastasis

Data for SLN detection are in the following sequence: Indocyanine green hotspot (P, Positive; N, Negative)/magnetic field during laparoscopic surgery (μT)/magnetic field of the extracted lymph nodes (μT)/iron content (μg). SLN = Sentinel lymph node, ND = Not detected.

**Fig 6:**
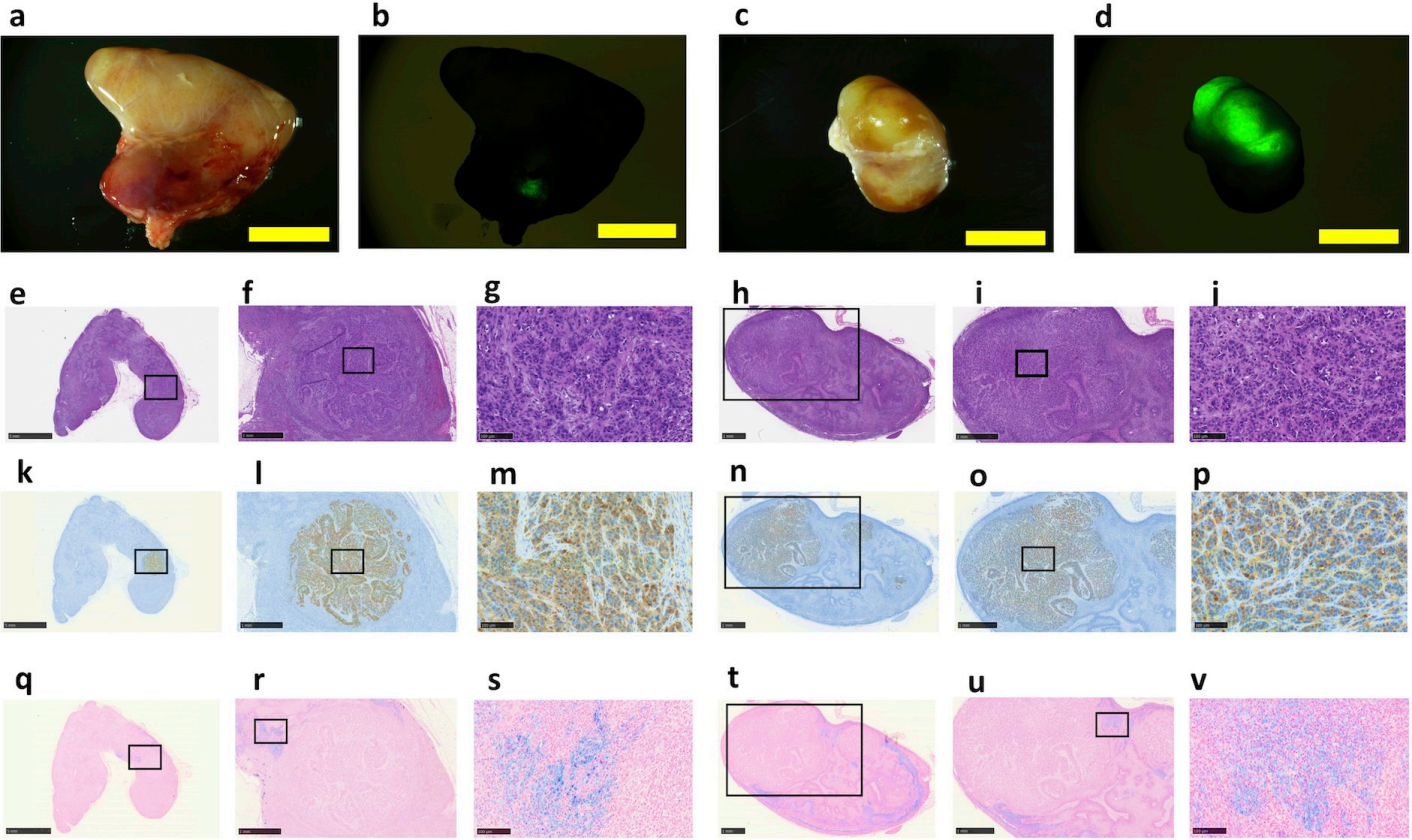
Fluorescence microscopy imaging and histological examinations of the two metastatic lymph nodes in the RAG2-knockout swine. (**a**) Bright-field view and (**b**) fluorescence microscopic view of the number 8 node. (**c**) Bright-field view and (**d**) fluorescence microscopic view of the number 13 node. (**e, f, g)** Haematoxylin and eosin staining of the number 8 node showing metastatic tumour cells. (**h, i, j**) Haematoxylin and eosin staining of the number 13 node showing metastatic tumour cells. (**k, l, m**) Immunohistochemical analysis of the number 8 node and (**n, o, p**) number 13 node using an anti-green fluorescent protein (GFP) antibody. (**q**, **r, s**) Iron staining of the number 8 node and (**t, u, v**) number 13 node. Scale bar = 5 mm (**a**–**d**, **e**, **k**, **q**), 1 mm (**f**, **h**, **i**, **l**, **n**, **o**, **r**, **t**, **u**) and 100 μm (**g**, **j**, **m**, **p**, **s**, **v**).

We resected all regional lymph nodes and examined them using a stereoscopic fluorescence microscope to confirm that the GFP signal was related to the cancer cells. We identified lymph node metastases in two lymph nodes: numbers 8 and 13 (Table 3 and Fig 6A–6V). We identified GFP hotspots more often in lymph node 13 than in node 8 (Fig 6B and 6D), and histological evidence of cancer metastasis was found in the same two lymph nodes (Fig 6E–6J). Immunohistochemistry using an anti-GFP antibody confirmed the presence of tumour cells (Fig 6K–6P), and iron staining revealed more iron deposition in node number 13 than in node number 8, which was also true for the tumour cells in these nodes (Fig 6Q–6V).

## Discussion

In this study, we found that detecting the SLNs of gallbladder cancer using a dual tracer laparoscopic technique with ICG and SPIO was feasible in a swine model. ICG showed a visual lymph flow map, and SPIO more accurately identified SLNs, similar to the RI tracer. These data are from animal experiments; however, detecting SLNs within 15 minutes following tracer injection is acceptable for laparoscopic surgery in humans. The weight, length and shape of the hand-held laparoscopic probe made it easy to use during the operation.

A magnetic tracer method has been used in patients with breast cancer and melanoma [14,29] and is reportedly not inferior to an RI technique regarding the rate of SLN detection. In gallbladder cancer, the SPIO method for SLN detection is likely preferable to an RI method because the RI injection 2 to 24 hours [7] prior to hepatobiliary surgery is unrealistic.

We modified a previously described Hall sensor probe [20] to generate a cordless laparoscopic magnetic probe for this study. In our experiments with swine, this probe enabled the accurate detection of SLNs *in situ*, and consistent results were observed *ex vivo* (Table 2). A previous study reported the *in situ* identification of SLNs in human stomachs using a laparoscopic gamma probe [30]. However, the shine-through phenomenon was reported to occur in RI procedures. The magnetic probe in this study was equipped with a sensor precisely positioned at the magnetic null point, as reported for magnetic handheld probes [20], which enabled highly sensitive measurements. The range of our probe is 1 cm ahead (Fig 2C), which prevents interference by steel instruments in the operating room or the shine-through phenomenon from the injection site. The probe has a sufficiently narrow detection range, can assess single lymph nodes and does not interfere with other nodes or the gallbladder injection site.

In this study, we used a dual tracer technique with ICG and SPIO. This dual tracer method is more sensitive in the detection of SLNs than ICG or SPIO alone. It is reportedly feasible to perform a single-tracer SLN procedure using NIR fluorescence ICG imaging in endometrial and gastric cancer laparoscopically [4,5]; however, 3 to 13 fluorescent lymph nodes were detected, rather than 1 or 2. The identified SLN number is also reportedly higher with the ICG method than the magnetic technique in patients with breast cancer [14]. The SLN is defined as the closest lymph node draining a tumour. ICG can pass quickly through the SLN to downstream lymph nodes; thus, using ICG alone can lead to unnecessary pathological assessments of multiple nodes. Although a single magnetic tracer is reportedly sufficient for SLN detection in breast cancer [14], brown-coloured SPIO nanoparticles in lymph nodes draining visceral organs cannot be visualised laparoscopically because the lymph nodes are surrounded by fatty tissue in adult humans. In this study, we first used ICG imaging to create a visual regional lymph node map and then assessed the magnetic fields using the probe according to the ICG hotspot information, resulting in the identification of one to two SLNs (S1 Video).

In this study, we established a RAG2-KO swine gallbladder cancer model with lymph node metastases to confirm that SLN mapping is effective under a tumour burden circumstance. In this swine model, we detected metastasis to two lymph nodes (numbers 8 and 13) by fluorescence microscopy and histological examination (Fig 6); however, the SPIO method identified metastasis to only a single node (number 13; Table 3). Because the number of tumour cells and the amount of iron were higher in the number 13 lymph node than the number 8 node, we interpreted these findings as indicating that the major SLN was the number 13 lymph node, and the number 8 lymph node was the minor SLN (Fig 6). Thus, in this model, cancer cell migration to the lymph node is quite similar to SPIO-detected lymphatic flow.

We found no iron-containing lymph nodes in one of five wild type swine (Swine 3; Table 2). There is a clinical association between subserosal invasion by gallbladder cancer and lymph node metastasis, and the subserosal layer of the gallbladder wall has a rich lymphatic network. However, there was less iron in the subserosal layer of the gallbladder in Swine 3 than in the other injected gallbladders (Fig 5). Thus, adequate subserosal injection of SPIO is necessary to detect gallbladder SLNs. The ICG tracer spread normally in Swine 3, supporting our contention that using dual tracers is preferable to using only one tracer.

The SPIO tracer used was Resovist®, which is a hydrophilic colloidal solution consisting of superparamagnetic iron oxide coated with carboxydextran developed by Schering AG. Resovist® is a clinically approved SPIO for contrast-enhanced magnetic resonance imaging of the liver. Safety data for the intravenous injection of Resovist® were extensively evaluated during clinical phases I–III, and toxic events caused by injected SPIOs are uncommon [31]. However, skin pigmentation and residual iron in the breast were the main side effects of local injection in breast cancer patients [21]. Our method involved local injection to the gallbladder. The SPIO was distributed to the interstitial connective tissue in all injected gallbladders and did not cause tissue inflammation or cell death, at least at the time of surgical extractions performed within 2 hours after SPIO injections in our histological examination. A previous study showed that the SPIO signal in SLNs is detectable within 30 min and retained for at least 4 hours in the nodes of breast cancer patients [32]. In addition, SPIO localization to higher echelon nodes beyond the SLN could occur 24 hours later [32]. In our study, the SPIO signal was confirmed to be retained in SLNs, at least until lymph node excision performed within one to two hours after the injection. Injected SPIO is known to be entrapped by macrophages in the lymphatic drainage system. For example, intravenously absorbed SPIO is reported to be captured by liver Kupffer cells and inflammatory cells from the reticuloendothelial system. Subsequently, SPIO is metabolized and regulated by iron homeostatic mechanisms [33].

Gallbladder cancer is increasingly being treated laparoscopically [34], and laparoscopic identification of the SLN for the gallbladder has great potential to resolve clinical questions. One of the unsolved issues is whether lymphadenectomy is necessary for patients with early gallbladder cancer. Although the closest lymph node to the gallbladder is the cystic node (number 12c), this node is sometimes bypassed by cancer cells [35]. The reported incidence of lymph node metastasis is 10.9% or less in patients with early gallbladder cancer [8]; thus, 90% of them do not need to undergo extensive lymphadenectomy by SLN investigation in the future. Further investigation of SLNs may be useful for clarifying lymph node mapping in gallbladder cancer.

A limitation of this study is that although the swine model is a good preclinical model, lymphatic flow around the gallbladder is very complex in humans and may differ from that in swine. Clinical trials are needed to establish a reliable means of identifying the SLNs of gallbladder cancers.

## Conclusion

In conclusion, our dual tracer method with ICG and SPIO using a laparoscopic magnetic probe to detect the SLNs of gallbladders in healthy wild-type swine and an established RAG2-KO swine gallbladder cancer model *in situ* is feasible. This study provides a potential bridge to the clinical adaptation of our laparoscopic method for detecting SLN metastasis from cancers of visceral organs, which are difficult to approach from the surface of the body or by endoscopy. Our procedure can enable the identification of SLNs for almost all intraabdominal organs that are laparoscopically accessible.

## Supporting information

S1 Video (MP4)
